# Analysis of the current situation of college students’ achievement motivation and influencing factors—an empirical analysis based on a college in Shandong Province

**DOI:** 10.3389/fpsyg.2025.1636209

**Published:** 2025-12-19

**Authors:** Shiying Dang, Yan Jin, Xingmeng Niu, Junyu Wang, Wenpei Yu, Weijun Zhou, Hao Sun, Hui Xie

**Affiliations:** 1 School of Clinical Medicine, Jining Medical University, Jining, Shandong, China; 2 School of Public Health, Shandong Second Medical University, Weifang, Shandong, China; 3 Affiliated Hospital of Jining Medical University, Jining, Shandong, China

**Keywords:** achievement motivation, college students, current status, empirical analysis, influencing factors

## Abstract

**Background:**

College students’ academic success and future growth are greatly impacted by achievement motivation, an innate desire for excellence. Higher education is an important time for students to socialize, and achievement motivation has a big impact on personal values, which is important for producing top-notch workers.

**Objective:**

The purpose of this study was to examine the current state of college students’ achievement motivation and the elements that influence it.

**Methods:**

2,849 students from a university in Shandong Province participated in a cross-sectional survey. The Achievement Motivation Scale was used to measure the motivation to strive for achievement vs. the motivation to avoid failure. SPSS version 27.0 was utilized for conducting descriptive statistics, single-factor analysis, and performing multiple linear regression analysis.

**Results:**

College students’ average achievement motivation score was 4.38 ± 12.99, with a noticeably unequal distribution across motivation levels. Gender, parental literacy level, personal and parental health condition, family annual income, future goals, and short-term plans all showed statistically significant variations in achievement motivation, according to a single-factor analysis (*p* < 0.05). However, multiple linear regression analysis showed that the direct effects of variables like family annual income and parental literacy level were no longer significant after adjusting for inter-variable interactions.

**Conclusion:**

This study confirms that gender, personal health condition, and short-term plans are important influencing factors and demonstrates considerable individual differences in university students’ achievement motivation with an uneven overall distribution. It offers both practical advice and theoretical foundations for applying differentiated instruction in higher education settings.

## 1 Introduction

The internal psychological drive that a person develops and sustains when confronted with worthwhile and significant goals is known as achievement motivation, and it is focused on the pursuit of success (Daumiller and Zarrinabadi, 2021). The achievement motivation theory states that “pursuing success” and “avoiding failure” are the two comparatively independent and opposing elements that make up this driving force (Brunstein and Heckhausen, 2025; Shang et al., 2023; Ehrlich and Bipp, 2016). The variations in a person’s choice of tasks, goal-setting, and degree of perseverance are determined by the relative intensity of the two. People who have high achievement motivation typically establish lofty objectives and put forth a lot of effort to achieve them. On the other hand, those with low achievement motivation may lack tenacity and perseverance and frequently select more easily attainable goals. People go through a transitional period during the university stage that is marked by slow psychological development, but they have not yet attained emotional and cognitive maturity. The creation of their work identity, the reconstruction of their value system, and the adjustment to a new social role are three crucial developmental tasks. Through mechanisms like self-efficacy, goal orientation, and career planning, the degree of achievement motivation indirectly affects post-graduation social adaption and inventive capacity in addition to directly influencing academic engagement and professional performance (Lu et al., 2024). As a result, higher education institutions must prioritize the development of achievement motivation in order to fulfill their objective of supporting student growth and long-term success.

Numerous elements, such as physical and mental health, family socioeconomic status, school education, social culture, self-efficacy, academic performance, and location of origin, are intimately linked to achievement motivation, according to research (Michou et al., 2014; Yang and Jiang, 2010; Butera et al., 2024). Specifically, the phenomenon of “poverty reinforcing achievement motivation” and the cross-cultural applicability of the four-element model of achievement motivation in the context of Chinese culture were confirmed in a large-sample study by Zeng et al. that used the latent profile analysis method for the first time (Zeng and Tu, 2024). This has significantly advanced the study of achievement motivation in China and supplied extremely useful empirical data for further research. This study focuses primarily on the one-dimensional influence of economic considerations on achievement motivation, despite its extensive scope and novel technique. In contrast, our study aims to develop a more multifaceted and thorough analytical framework for the factors influencing achievement motivation by thoroughly examining the impact of four core variables on achievement motivation: individual characteristics, family background, school education, and social environment. Furthermore, this study’s participants are first-year students. The development of this group’s achievement motivation has greater plasticity and research worth because they are just entering the university stage.

In order to improve college students’ achievement motivation, provide theoretical support and practical references for higher education management, mental health education, and future planning guidance, and ultimately promote the all-around development of college students as well as social harmony and progress, this study uses a cross-sectional survey design based on the theory of achievement motivation.

## 2 Materials and methods

### 2.1 Study population

This cross-sectional study was conducted from September to December 2024 among college students at a university in Shandong Province, utilizing a convenience whole cluster sampling method. The inclusion criteria were: (1) full-time college students formally enrolled in a university in Shandong Province; (2) A freshman of the class of 2024; (3) voluntary participation and signing of informed consent. The exclusion criteria were: (1) unofficially registered students; (2) students who were unable to complete the questionnaire due to physical or mental illness; (3) those who completed invalid questionnaires. A total of 3,201 questionnaires were distributed and 2,849 valid questionnaires were recovered, with a valid response rate of 89.0%. Before the survey, the staff was professionally trained, and during the survey, the students were introduced to the purpose, significance, and confidentiality principles to ensure that the questionnaires were independently completed and recovered promptly. The online survey was sent to class groups and other channels with a link to the questionnaire star, and a person was assigned to answer questions and remind students to complete the questionnaire regularly. Surveyors were trained uniformly to clarify the process and requirements to ensure that the questionnaire was designed concisely and clearly and that the survey site was strictly managed to resolve questions promptly and prevent students from answering randomly. During the data entry phase, we used two-person, dual-computer entry with strict calibration to ensure that the data were accurate and complete. During data analysis, we reviewed and cleaned the data again, eliminating outliers and erroneous data to ensure the reliability of the analysis results.

### 2.2 Questionnaire design

#### 2.2.1 Self-organized personal information sheet

In the design process of this self-compiled personal information form, multiple elements that affect college students’ achievement motivation were fully considered. The basic information of college students was collected through questionnaire surveys. This study collected information from four dimensions: individual (gender, age, personal health condition), family (parents’ health condition, family structure), school (short-term plans), and society (future goals, family annual income, parents’ literacy level, living expenses), and obtained data through students’ self-reports. This approach can more comprehensively and deeply explore the influencing factors of college students’ achievement motivation, providing rich and valuable data support for subsequent analysis and research.

#### 2.2.2 Achievement motivation scale

Achievement Motivation Scale was developed by Norwegian psychologists Gjesme, T. and Nygard, R. in 1970 and has been revised several times and gradually improved (Hermans, 1970). The Chinese version was translated by our researcher Ye Renmin in collaboration with Hegtvet, K. A. from Norway in 1988 and revised in 1992 in a sample of college and middle school students (Ye and Kh, 1992). Its use as a more mature scale for assessing individual achievement motivation characteristics and levels has been widely used in China (Elliot and Sommet, 2023). This scale contains 30 questions and is scored on a 4-point Likert scale from 1 (not at all) to 4 (very much): (1) “not at all” (2) “somewhat not at all” (3) “mostly at all “(4) “Very much in line.” The results of this scale, which measures both dimensions of achievement motivation, including Motivation to seek success and Motivation to avoid failure subscales (15 questions each), are more conducive to predicting an individual’s behavior, compared to a scale that only measures overall achievement motivation (Fan and Zhang, 2009). The achievement motivation score consists of the Motivation to seek success score minus the Motivation to avoid failure score, with higher scores indicating greater achievement motivation (Hermans, 1970).

### 2.3 Statistical methods

In this study, SPSS software was used to enter, organize, and analyze the collected data. All survey data were entered into SPSS 27.0, an internal consistency test was performed on the achievement motivation scale, and descriptive statistics, one-way and regression analysis were performed on the variables, where *p* < 0.05 indicated that the difference was statistically significant.

## 3 Results

### 3.1 Reliability and validity test

#### 3.1.1 Reliability

The reliability of the achievement motivation scale was gauged by investigating its internal concordance reliability, which was determined using Cronbach’s alpha coefficient (see Table 1 for details). The analysis indicates that the overall internal consistency coefficient of the Achievement Motivation Scale is 0.782, suggesting that the scale possesses high reliability, substantial internal consistency, and the capacity to reliably and validly reflect the genuine thoughts and sentiments of the respondents, thereby ensuring the reliability and validity of the research outcomes.

**Table 1:** Reliability analysis of the achievement motivation scale.

Reliability index	Numerical value
Cronbach’s alpha coefficient	0.782
Standardized Cronbach’s alpha coefficient	0.782

The reliability coefficients for the scale are greater than the recommended threshold of 0.70, indicating good internal consistency and meeting psychometric requirements.

#### 3.1.2 Validity

The validity analysis of this scale was validated using KMO and Bartlett’s test (Table 2), which yielded a KMO value of 0.945 (KMO > 0.8), the study data were well suited for extracting the information (which side by side reflects the validity was very good) and Bartlett’s test of sphericity *p* < 0.05, which indicates that the data of the questionnaire has good validity (Deng et al., 2018).

**Table 2:** Validity analysis of the achievement motivation scale.

Inspection object	KMO	Bartlett Text		
		Approximate chi-square (math.)	df	sig
Achievement motivation scale	0.94	38626.303	435	<0.001

A KMO value greater than 0.9 indicates that there are many common factors among the variables, making factor analysis suitable. Bartlett’s sphericity test Sig. < 0.001, indicating that the scale data follow a multivariate normal distribution and that factor analysis has good applicability.

### 3.2 Common method bias assessment

Since all the data in this study were collected via self-administered questionnaires, there may be common method bias. Therefore, this study employed the method of controlling for the effects of an unmeasured latent methods factor technique for testing purposes (Tan and Yun, 2020). The results indicated that the baseline measurement model exhibited an acceptable fit (χ^2^/df = 9.818, CFI = 0.913, TLI = 0.900, RMSEA = 0.056). After introducing the common method factor, the model fit indices showed no significant improvement (χ^2^/df = 9.952, CFI = 0.911, TLI = 0.898, RMSEA = 0.056; ΔCFI < 0.01, ΔRMSEA < 0.01). This suggests that common method bias is not a substantial concern in this study (Cheung and Rensvold, 2002; Podsakoff et al., 2003). This study has good structural validity and can provide reliable data support for subsequent analysis.

### 3.3 Achievement motivation score

This study shows the basic distribution of achievement motivation among university students based on the analysis of data from 2,849 valid questionnaires (detailed results are provided in Tables 3, 4). Statistics showed that the sample’s overall achievement motivation score was 4.38 ± 12.99 points (minimum −42 points, maximum 45 points). Although more than half (59.00%) of the students exhibited good achievement motivation inclinations, more than one-third (36.12%) of the students received scores below zero. This implies an uneven distribution of achievement motivation scores among university students. The relatively high degree of dispersion in achievement motivation scores indicates that there is significant individual variance and group heterogeneity within the sample.

**Table 3:** Overall level of achievement motivation.

Variable	X min	X max	M ± SD
Achievement motivation score	−42.00	45.00	4.38 ± 12.99
Motivation to avoid failure	15.00	60.00	35.49 ± 8.10
Motivation to seek success	15.00	60.00	39.87 ± 7.60

The achievement motivation score consists of the motivation to seek success score minus the motivation to avoid failure score, with higher scores indicating greater achievement motivation.

**Table 4:** Frequency distribution of achievement motivation scores (*n* = 2,849).

Variable	Score range	*N* (%)
Achievement motivation score	>0	1,681 (59.00%)
	0	139 (4.88%)
	<0	1,029 (36.12%)

The achievement motivation score consists of the motivation to seek success score minus the motivation to avoid failure score.

### 3.4 Descriptive statistics

A total of 3,201 questionnaires were distributed and 2,849 valid questionnaires were returned, of which 1,536 (53.9%) were female and 1,313 (46.1%) were male, with a mean age of 18.98 ± 1.21 years, and the highest percentage of students in the ≤18 years age group was 67.1% (1912). The rest of the demographics are detailed in Table 5.

**Table 5:** Descriptive analysis of basic demographic characteristics (*n* = 2,849).

Items	Option	*N* (%)
Gender	Male	1,313 (46.09)
	Female	1,536 (53.91)
Age	18 years and under	1912 (67.11)
	Over 18 years old	937 (32.89)
Father’s literacy level	Primary school and below	265 (9.30)
	Junior high school	954 (33.49)
	High School	599 (21.02)
	College	483 (16.95)
	Undergraduate	468 (16.43)
	Master’s degree and above	80 (2.81)
Mother’s literacy level	Primary school and below	433 (15.20)
	Junior high school	957 (33.59)
	High School	526 (18.46)
	College	497 (17.44)
	Undergraduate	394 (13.83)
	Master’s degree and above	42 (1.47)
Personal health condition	Not sick	2,353 (82.59)
	Sick	495 (17.37)
Father’s health condition	Not sick	1919 (67.36)
	Sick	930 (32.64)
Mother’s health condition	Not sick	2,164 (75.96)
	Sick	685 (24.04)
Family structure	Multi-child	2035 (71.43)
	Only child	814 (28.57)
Family annual income	Equal or less than 10,000 yuan	157 (5.51)
	10,001 ~ 30,000 yuan	435 (15.27)
	30,001 ~ 50,000 yuan	488 (17.13)
	50,001 ~ 80,000 yuan	343 (12.02)
	80,001 ~ 100,000 yuan	413 (14.50)
	100,001 ~ 150,000 yuan	554 (19.45)
	more than 150,00 yuan	459 (16.11)
Living expenses	Equal or less than 1,000 yuan	230 (8.07)
	1,001 ~ 1,500 yuan	1,278 (44.86)
	1,501 ~ 2,000 yuan	1,105 (38.79)
	More than 2,000 yuan	236 (8.28)
Future goals	No direction	50 (1.80)
	Employment	319 (11.20)
	Civil servants	95 (3.30)
	Postgraduate	2,361 (82.90)
	Other	24 (0.80)
Short-term Plan	No goal	94 (3.30)
	Graduate without failing a class	1,523 (53.46)
	Run for the class committee or student organization	913 (32.05)
	Work hard to finish school and get a scholarship	1851 (64.97)
	Participate in clubs or other non-major programs	856 (30.05)
	Participate in competitions in your major	858 (30.12)
	Others	176 (6.18)

The sum of some classification frequencies may be slightly rounded and not equal 100%, which is within the statistical allowable rang.

### 3.5 Single-factor analysis

An individual’s psychological characteristics and behavioral habits are thought to be directly linked to achievement motivation. This study used the one-way analysis of variance method to investigate the impact of demographic factors on college students’ achievement motivation, including gender, parents’ literacy level, parents’ physical and personal health condition, family annual income, future goals, and short-term plans. The findings demonstrate that a variety of factors, including gender, personal health condition, parents’ health condition, future goals, and short-term plans, have a substantial impact on college students’ level of accomplishment motivation. In particular, all aspects of achievement motivation exhibit highly significant gender differences (*p* < 0.001), with a moderate impact size. When it comes to overall achievement motivation and the motivation to seek success, males are much more motivated than women. One important determining aspect is one’s own state of health. All motivation characteristics show significant differences between sick and non-sick college students (*p* < 0.001), with the biggest impact size on the motivation to avoid failure (Cohen’s d = 0.263). In the meantime, college students’ motivation to seek success and overall achievement motivation was also significantly impacted by their parental health condition, with a moderate effect size (*p* < 0.001, Cohen’s d > 0.139). When it comes to goal-setting, well-defined short-term plans significantly improve all aspects of achievement motivation (*p* < 0.001), whereas future goals only significantly affect the motivation to seek success and the overall achievement motivation; they have no significant effect on the motivation to avoid failure.

It is important to note that while parents’ literacy level and family annual income exhibit statistical significance in some dimensions (*p* < 0.05), their effect size indicators (η^2^ < 0.01) show that these factors’ actual explanatory power for the variation in achievement motivation is relatively limited. On the other hand, achievement motivation did not significantly alter depending on parameters like age, family structure, or monthly living expenses. This implies that we should focus more on goal-oriented advice, psychological support, and individual health management in the intervention strategies to improve college students’ accomplishment motivation. Table 6 provides specific information on the pertinent facts.

**Table 6:** Analysis of variance in achievement motivation for general demographic information.

Items	Options	Motivation to seek success				Motivation to avoid failure				Achievement motivation			
		M ± SD	t/F	*p*	η^2^/Cohen’s d	M + SD	t/F	*p*	η^2^/Cohen’s d	M + SD	t/F	*p*	η^2^/Cohen’s d
Gender	Male	40.67 ± 7.89	5.182	<0.001	0.196	34.86 ± 8.42	−3.82	<0.001	0.145	5.81 ± 13.16	5.457	<0.001	0.205
	Female	39.19 ± 7.28				36.03 ± 7.78				3.16 ± 12.71			
Age	18 years and under	39.93 ± 7.39	0.536	0.592	0.022	35.26 ± 7.95	−2.174	0.03	0.087	4.66 ± 12.90	1.678	0.093	0.067
	Over 18 years old	39.76 ± 8.02				35.96 ± 8.37				3.79 ± 13.15			
Father’s literacy level	Primary school and below	39.51 ± 7.62	3.083	0.009	0.005	35.96 ± 7.99	2.199	0.052	0.004	3.55 ± 12.39	3.437	0.004	0.006
	Junior high school	39.47 ± 7.48				36.01 ± 7.87				3.46 ± 12.63			
	High School	39.77 ± 7.64				35.02 ± 8.18				4.76 ± 12.84			
	College	39.74 ± 7.42				35.61 ± 7.91				4.12 ± 13.12			
	Undergraduate	40.89 ± 7.62				34.78 ± 8.15				6.12 ± 13.01			
	Master’s degree and above	41.43 ± 8.95				34.90 ± 10.74				6.53 ± 17.50			
Mother’s literacy level	Primary school and below	38.73 ± 7.92	3.516	0.004	0.006	36.52 ± 7.98	2.459	0.031	0.004	2.21 ± 13.02	4.126	0.001	0.007
	Junior high school	40.02 ± 7.31				35.27 ± 7.95				4.75 ± 12.47			
	High School	39.65 ± 7.30				35.46 ± 8.15				4.19 ± 12.68			
	College	40.00 ± 7.89				35.75 ± 7.97				4.25 ± 13.41			
	Undergraduate	40.71 ± 7.67				34.68 ± 8.23				6.07 ± 13.22			
	Master’s degree and above	41.36 ± 8.98				35.10 ± 11.12				6.26 ± 17.49			
Personal health condition	Not sick	39.00 ± 7.97	−2.791	<0.001	0.138	37.24 ± 8.17	5.311	<0.001	0.263	1.76 ± 13.50	−4.949	<0.001	0.245
	Sick	40.05 ± 7.51				35.13 ± 8.04				4.92 ± 12.81			
Father’s health condition	Not sick	40.34 ± 7.60	−4.792	<0.001	0.191	35.38 ± 8.04	1.084	0.279	0.043	4.96 ± 12.80	−3.476	0.001	0.139
	Sick	38.89 ± 7.50				35.73 ± 8.22				3.16 ± 13.28			
Mother’s health condition	Not sick	38.83 ± 7.44	−4.134	<0.001	0.181	36.35 ± 7.87	3.170	0.002	0.139	2.48 ± 12.50	−4.400	<0.001	0.193
	Sick	40.20 ± 7.62				35.22 ± 8.16				4.98 ± 13.08			
Family structure	Multi-child	40.27 ± 8.14	1.696	0.09	0.073	35.10 ± 8.46	−1.649	0.099	0.068	5.17 ± 13.88	1.981	0.048	0.086
	Only child	39.71 ± 7.37				35.65 ± 7.95				4.06 ± 12.60			
Family annual income	Equal or less than 10,000 yuan	39.80 ± 8.45	6.146	<0.001	0.013	35.45 ± 8.56	3.011	0.006	0.006	4.35 ± 13.09	6.245	<0.001	0.013
	10,001 ~ 30,000 yuan	38.91 ± 7.38				36.38 ± 7.75				2.53 ± 12.72			
	30,001 ~ 50,000 yuan	39.17 ± 7.48				35.95 ± 7.86				3.22 ± 12.04			
	50,001 ~ 80,000 yuan	39.18 ± 7.21				35.67 ± 7.72				3.52 ± 12.71			
	80,001 ~ 100,000 yuan	39.90 ± 7.56				35.83 ± 7.88				4.07 ± 12.95			
	100,001 ~ 150,000 yuan	40.34 ± 7.12				34.92 ± 7.68				5.42 ± 12.45			
	more than 150,00 yuan	41.47 ± 8.23				34.45 ± 9.28				7.03 ± 14.39			
Living expenses	equal or less than 1,000 yuan	39.77 ± 7.91	1.272	0.282	0.001	36.04 ± 7.92	1.421	0.235	0.001	3.72 ± 13.20	1.679	0.169	0.002
	1,001 ~ 1,500 yuan	39.60 ± 7.32				35.60 ± 7.84				4.00 ± 12.34			
	1,501 ~ 2,000 yuan	40.09 ± 7.65				35.46 ± 8.11				4.64 ± 13.26			
	more than 2,000 yuan	40.41 ± 8.49				34.58 ± 9.48				5.83 ± 14.71			
Future Goals	No direction	37.00 ± 10.24	6.781	<0.001	0.009	36.64 ± 10.03	1.098	0.356	0.002	0.36 ± 15.76	4.500	0.001	0.006
	Employment	38.24 ± 7.88				35.98 ± 8.03				2.26 ± 13.07			
	Civil servants	40.66 ± 8.28				35.33 ± 8.44				5.34 ± 14.72			
	Postgraduate	40.10 ± 7.41				35.43 ± 8.04				4.66 ± 12.81			
	Other	42.08 ± 9.24				33.13 ± 9.17				8.96 ± 15.78			
Short-term Plan	No goal	39.36 ± 10.13	17.591	<0.001	0.042	36.67 ± 10.06	8.167	<0.001	0.020	2.69 ± 15.16	17.756	<0.001	0.042
	Graduate without failing a class	39.00 ± 7.50				36.18 ± 7.90				2.82 ± 12.83			
	Run for the class committee or student organization	41.45 ± 7.25				33.93 ± 8.28				7.51 ± 12.95			
	Work hard to finish school and get a scholarship	40.87 ± 7.15				34.85 ± 7.96				6.02 ± 12.61			
	Participate in clubs or other non-major programs	41.35 ± 7.15				34.50 ± 8.14				2.69 ± 15.16			
	Participate in competitions in your major	42.44 ± 7.35				33.79 ± 8.26				8.65 ± 13.20			
	Others	41.06 ± 7.96				35.04 ± 8.03				6.02 ± 14.55			

The t value is used for binary categorical variables (such as gender), and the F value is used for multi-categorical variables (such as educational level); η^2^ (effect size) is used for multi-categorical variables, while Cohen’s d is used for binary categorical variables. The larger the value, the stronger the impact of the differences between groups. Significance annotation: *p* < 0.05 indicates a statistically significant difference, *p* < 0.01 indicates a highly statistically significant difference, and *p* < 0.001 indicates an extremely highly statistically significant difference.

### 3.6 Multiple linear regression analysis

Achievement motivation was designated as the dependent variable in this investigation. A multiple linear regression model was created using all the variables that demonstrated statistical significance (*p* < 0.05) in the univariate analysis, including gender, parental literacy level, personal health condition, parental health condition, family annual income, future goals, and short-term plans. Gender, personal health condition, and short-term plans emerged as significant predictors of college students’ achievement motivation after adjusting for other demographic characteristics, according to the multiple linear regression analysis results (see Table 6). In particular, female students showed considerably lower levels of achievement motivation than male students (*β* = −0.132, *p* < 0.001). In a similar vein, pupils who reported illness showed considerably less achievement motivation than those who did not (*β* = −0.064, *p* < 0.001). With regard to short-term plans, using “no goals for the time being” as the reference category, the following categories were found to positively predict achievement motivation: “strive to complete studies and obtain scholarships” (*β* = 0.082, *p* < 0.001), “participate in clubs or non-professional projects” (*β* = 0.087, *p* < 0.001), “take part in professional competitions” (*β* = 0.042, *p* < 0.05), and “other” plans (*β* = 0.145, *p* < 0.001). In contrast, “running for class committee” was associated with a significant negative predictive effect (*β* = −0.103, *p* < 0.001). According to these results, college students’ achievement motivation can be significantly increased by male gender, good health, and having positive short-term plans (such as running for class council, finishing school to receive a scholarship, or participating in clubs or non-professional programs). On the other hand, being female, being ill, and having the short-term plan of “avoiding course failure” are risk factors for achievement motivation and may have a detrimental effect on it.

Notably, when included in the multivariate regression model, variables like paternal literacy level, parental health condition, family annual income, and future goals did not show significant predictive effects compared to the single-factor analysis results. Only the “junior high school” category showed a slightly favorable predictive effect when compared to the reference group of “primary school or below” in terms of Mother’s literacy level (For more information, see Table 7).

**Table 7:** Multifactor analysis of achievement motivation among college students^a^.

Items	Options	B	S. D	β	t	*p*	Tolerances	VIF
Gender	Male (comparison group)	0						
	Female	−3.441	0.472	−0.132	−7.294	<0.001	0.952	1.05
Father’s literacy level	Primary school and below (comparison group)	0						
	Junior high school	−1.138	0.891	−0.041	−1.277	0.202	0.298	3.36
	High School	−0.143	0.997	−0.004	−0.143	0.886	0.319	3.133
	College	−1.283	1.102	−0.037	−1.165	0.244	0.309	3.24
	Undergraduate	−0.093	1.167	−0.003	−0.08	0.936	0.282	3.552
	Master’s degree and above	0.64	1.827	0.008	0.35	0.726	0.578	1.729
Mother’s literacy level	Primary school and below (comparison group)	0						
	Junior high school	1.891	0.751	0.069	2.517	0.012	0.418	2.391
	High School	0.643	0.907	0.019	0.709	0.478	0.425	2.353
	College	0.152	0.99	0.004	0.153	0.878	0.374	2.676
	Undergraduate	1.016	1.098	0.027	0.926	0.355	0.367	2.728
	Master’s degree and above	0.171	2.27	0.002	0.075	0.94	0.704	1.421
Personal health condition	Not sick (comparison group)	0						
	Sick	−2.205	0.632	−0.064	−3.491	<0.001	0.919	1.088
Father’s health condition	Not sick (comparison group)	0						
	Sick	−0.361	0.545	−0.013	−0.663	0.508	0.808	1.238
Mother’s health condition	Not sick (comparison group)	0						
	Sick	−1.333	0.598	−0.044	−2.227	0.026	0.805	1.242
Family annual income	Equal or less than 10,000 yuan (comparison group)	0						
	10,001 ~ 30,000 yuan	−1.66	1.151	−0.046	−1.443	0.149	0.308	3.247
	30,001 ~ 50,000 yuan	−1.278	1.134	−0.037	−1.127	0.26	0.288	3.468
	50,001 ~ 80,000 yuan	−1.087	1.2	−0.027	−0.906	0.365	0.345	2.897
	80,001 ~ 100,000 yuan	−0.555	1.173	−0.015	−0.474	0.636	0.309	3.237
	100,001 ~ 150,000 yuan	0.439	1.146	0.013	0.383	0.702	0.256	3.908
	More than 150,00 yuan	1.716	1.18	0.049	1.454	0.146	0.28	3.575
Future goals	No direction (comparison group)	0						
	Employment	−1.883	1.971	−0.019	−0.955	0.339	0.786	1.273
	Civil servants	1.185	1.445	0.016	0.82	0.412	0.783	1.277
	Postgraduate	0.205	0.752	0.006	0.273	0.785	0.656	1.525
	Other	3.491	2.61	0.025	1.337	0.181	0.925	1.081
Short-term plan	No goal (comparison group)	0						
	Graduate without failing a class	0.088	1.422	0.001	0.062	0.95	0.816	1.226
	Run for the class committee or student organization	−2.676	0.496	−0.103	−5.395	<0.001	0.86	1.162
	Work hard to finish school and get a scholarship	2.28	0.55	0.082	4.146	<0.001	0.8	1.25
	Participate in clubs or other non-major programs	2.377	0.564	0.087	4.211	<0.001	0.726	1.377
	Participate in competitions in Your major	1.178	0.559	0.042	2.109	0.035	0.803	1.245
	Others	4.117	0.571	0.145	7.211	<0.001	0.768	1.302

^a^Dependent variable: achievement motivation. The reference group is assigned a value of 0, and the regression coefficients for other classifications are calculated based on the reference group. When the tolerance is greater than 0.1 and the VIF is less than 10, it indicates that there is no severe multicollinearity among the variables and the stability of the regression model is good. The larger the absolute value of the standardized regression coefficient (Beta), the stronger the influence of this variable on achievement motivation. Significance annotation: *p* < 0.05 indicates a statistically significant difference, *p* < 0.01 indicates a highly statistically significant difference, and *p* < 0.001 indicates an extremely highly statistically significant difference.

## 4 Discussion

### 4.1 The current situation of achievement motivation

This study discovered that the general distribution of achievement motivation levels among university students is skewed, with significant inherent differentiation. This discovery is consistent with earlier research (Elliot et al., 2017; Spurk et al., 2020). The underlying causes of such inequalities are complicated and may be the consequence of a combination of factors such as individual characteristics, family environment, educational background, and social expectations (Howard et al., 2021). Furthermore, the phenomenon of differentiation in achievement motivation suggests that we should pay more attention to individual differences and implement targeted measures to stimulate students’ intrinsic motivation, assisting them in developing a positive achievement orientation and coping strategies. Students with a positive achievement motivation should be given assistance to retain their drive. For students with low achievement motivation or a dominant inclination to avoid failure, targeted assistance should include psychological therapy, goal reconstruction, and successful experiences. Future study should move beyond the broad pursuit of motivational levels and instead concentrate on the specific identification of discrete achievement motivation groups and their influencing elements, allowing for the creation of customized intervention strategies.

### 4.2 Influencing factors of achievement motivation

#### 4.2.1 Personal level

According to this study, accomplishment motivation is highly correlated with individual characteristics like gender and health. Significant gender variations in achievement motivation were revealed by the questionnaire survey results. Male students were more competitive and goal-oriented, whereas female students were more risk-averse and sensitive to failure. These findings are consistent with those of Kaura and Sharma (2015), who discovered that gender had a substantial impact on accomplishment motivation, potentially as a result of conventional social and cultural gender norms (Borgonovi and Han, 2021; Maheswari and Aruna, 2016). In particular, boys are typically expected to be more socially responsible and to be under more pressure to compete. Boys are raised to be the “backbone” of the family from an early age and are encouraged to take part in challenges and competitions due to parental attitudes. They are more motivated to succeed and feel more achievement motivation overall as a result of this type of upbringing (Yu et al., 2021; Hines, 2020). On the other hand, girls are more likely to be required to do household duties like childrearing and housekeeping, which may lessen their achievement motivation. This could lessen their drive to attain great achievement and make it simpler for them to put in a lot of effort to reach their own objectives (Guan et al., 2023).

Gender roles develop later in life, despite the fact that sex-related physical traits are intrinsic. Individuals’ achievement motivation can be effectively increased by understanding gender disparities, strengthening the positive aspects of gender roles, improving the development of health courses, and incorporating well-defined objectives, prompt feedback, and a supportive environment of short-term plans (Kavousipour et al., 2015). In schools, gender education should be customized to meet the needs of individual students. Gender education should be tailored to each student’s requirements in schools. To help girls overcome social restraints and prejudices, schools could, for instance, invite “successful women” and other well-known individuals to conduct special lectures. This would encourage their drive to succeed and enable them to pursue self-improvement without being constrained by conventional gender norms. Parents ought to reconsider gender-specific schooling and focus more on their kids’ individuality. In the new era, they should be democratic parents and encourage their kids to actively explore.

Achievement motivation is significantly impacted by health, which is a crucial aspect of college students’ overall quality (Mahdavi et al., 2023). In agreeing with the findings of Andersen et al. (2016), this study discovered that healthy college students typically have higher achievement motivation. Healthy college students are able to spend more time and energy to achievement-related activities like studying, conducting research, and engaging in social events. This increases their achievement motivation (Merkt and Gawrilow, 2016). College students with health issues, on the other hand, could be self-conscious and lack confidence, which lowers their achievement motivation (Medlicott et al., 2021). As a result, schools ought to prioritize the health of their kids. They can support students in maintaining a healthy physical and mental state and increase their drive for success by providing mental health education courses and planning physical exercise activities. At the same time, parents should respect their children’s individuality and viewpoints, give them all the attention and support they need, and cultivate a cozy, peaceful, and democratic home environment. Children will be more self-assured and driven to achieve their goals when they experience the love and support of their family (Li et al., 2022; Meng et al., 2025).

#### 4.2.2 Family and school level

Multiple types of short-term goals have distinct effects on achieving motivation, according to research. Achievement motivation is significantly positively predicted by goals that center on developing one’s own skills and pursuing personal interests (e.g., “striving for scholarships,” “participating in clubs or non-professional projects,” “taking part in professional competitions”). Nonetheless, there is a strong negative predictive effect in the “election for class committee positions” with considerable social hazards and assessment pressure (Atkinson, 1957; McClelland et al., 1953). This finding highlights the impact of target content attributes on motivation. While performance objectives that highlight social assessment may trigger students’ need to avoid failure, so impeding the development of their achievement motivation, mastery goals that emphasize ability growth can effectively boost achievement motivation (Ryan and Deci, 2000; Bögels and Mansell, 2004; Chen and Shi, 2015). As a result, schools should intentionally encourage students to concentrate on their own personal development and develop a goal-oriented mindset that is focused on gaining knowledge and skills during the academic coaching process.

#### 4.2.3 Social level

The findings demonstrate that college students’ overall accomplishment motivation and its two aspects are significantly impacted by the family income level, which is a crucial indicator of the family’s socioeconomic standing. In particular, compared to their classmates from middle-income (50,000–150,000 yuan) and low-income (<50,000 yuan) homes, students from families with an annual income surpassing 150,000 yuan exhibit noticeably greater levels of motivation to seek success and overall achievement motivation. This result is in line with earlier international research (Adsul et al., 2008; Sommet et al., 2019). This conclusion, however, is at odds with Zeng et al.’s findings, which suggested that “poverty may enhance achievement motivation.” The following are the primary causes of this discrepancy: First, a significant contributing factor to the disparate outcomes could be the heterogeneity in research samples. Zeng et al.’s research subjects comprised university students from low-income families who had passed the Federal Grant Scheme review. This cohort exhibits strong positive selectivity, indicating that low-income university students inherently possess robust motivation for success. Our research sample also included ordinary undergraduates from diverse socioeconomic backgrounds who had not undergone similar selection processes. Family socioeconomic status exerted a more direct and significant influence on achievement motivation. Shandong, renowned as the ‘hometown of Confucius and Mencius, the land of learning,’ exhibits a strong awareness of investment in family education, particularly pronounced among high-income households. High-income families are more inclined to convert their financial resources into educational capital for their kids through foreign exchanges, quality growth, and after-school tutoring. Students’ self-efficacy and sense of success are greatly increased by these investments, which increases their achievement motivation (Hill, 2021). In contrast (Wang et al., 2021), students from poor families have a significantly higher motivation to avoid failure than those from high-income families. This might be because students from poor families face more economic pressure and uncertainty, and are more sensitive to stress, leading them to be more inclined to avoid risks and the negative impacts brought about by failure. Finally, different types of schools also vary in terms of educational objectives, teaching methods, etc., which will also have an impact on students’ achievement motivation. Therefore, the higher motivation of high-income students shows the ongoing good impact of family resources on motivation from middle school to college, while the higher motivation of poor students may be the psychological resilience effect during the college stage. The two show the situational and phased variations in the development of achievement motivation rather than being incompatible (Dweck and Leggett, 1988).

We suggest the following diversified family support strategies based on the results of this study, which are adapted to different socioeconomic circumstances (Mistry and Elenbaas, 2021). In particular, low-income families should place a high priority on offering their kids emotional support and encouragement in order to help them set positive objectives for their lives and lessen any negative feelings that can result from financial hardship. It is recommended that middle-class families concentrate on building their kids’ resilience and teaching them to keep a positive attitude when facing challenges in their academic and professional endeavors. In high-income households, it is essential to avoid imposing excessive academic or achievement pressure, allowing greater autonomy in personal development while prioritizing their psychological and physical well-being. By implementing such tailored approaches, it is possible to better address the distinct needs of students from diverse family income and facilitate their holistic development (Shabnam, 2025).

This study discovered that the direct impacts of variables like parents’ literacy level and family annual income on achievement motivation were no longer significant after adjusting for other variables. This finding is in line with earlier research’s conclusion that, as the primary component of family socioeconomic status, the influence of parents’ literacy level and family annual income typically has an indirect impact on achievement motivation through mediating factors (Tan et al., 2021). According to study by Caprara et al. (2008), students’ desire for achievement might be impacted by their family’s financial level through their feeling of self-efficacy. The direct impact of family socioeconomic status considerably diminishes after adjusting for self-efficacy. In particular, children from families with higher socioeconomic standing typically have greater access to resources, successful experiences, and role models, which helps them develop a stronger feeling of self-efficacy. One of the most direct motivators for accomplishment is self-efficacy. Parenting practices have also been shown to be a significant mediating factor in addition to self-efficacy (Su et al., 2015). In general, democratic and independent parenting approaches are more common among parents with greater education and less financial strain. This kind of positive parenting style can not only directly motivate individuals to pursue success, but also indirectly enhance their achievement motivation by promoting the development of their self-regulation ability and executive function (Luo et al., 2023). Furthermore, Haimovitz and Dweck (2017) research demonstrates that how parents perceive and respond to failure, whether as a learning opportunity or based on ability, has a substantial impact on their children’s thinking patterns. Parents with a high level of education are more likely to communicate a “growth mindset,” which indirectly boosts their children’s achievement motivation. Finally, family income and parents’ literacy level influence achievement motivation through a variety of mediating variables. Future study should investigate additional mediating variables and their mechanisms in order to acquire a more complete knowledge of the complicated relationship between family socioeconomic status and achievement motivation. In order to achieve more targeted and successful intervention, the function of these mediating variables should be thoroughly considered when developing educational policy and family support methods.

### 4.3 Limitations and future directions

Despite the fact that this study offers insightful information about the current state of college students’ achievement motivation and the elements that influence it, there are still a number of limitations that also indicate the route for future research. First, this study used cross-sectional survey data for its research design. It is challenging to determine the causal linkages between variables, even though it can show their associations. In order to better describe the temporal and causal relationships between individual traits, short-term plans, health conditions, and achievement motivation levels, future research should use longitudinal designs to monitor the dynamic trajectory of students’ achievement motivation from enrollment to graduation. Second, in terms of sample sources, the study’s data is focused in one area, and the samples’ representativeness is restricted, which somewhat compromises the research findings’ external validity. Future studies should perform multi-level and cross-regional comparative analyses, broaden the sample sources, and include students from other fields, geographies, and cultural backgrounds. Finally, while this study revealed several mediating processes when investigating the influence of characteristics such as family annual income and parental educational level on success motivation, more in-depth research and extension are required. Future research should use more rigorous research methodologies, delve further into the interaction paths and mediating mechanisms between distinct variables, and develop a more systematic knowledge of their overall influence patterns.

## 5 Conclusion

This study employs empirical analysis to demonstrate how the drive for achievement among college students varies greatly on an individual basis and is generally distributed unevenly. It provides theoretical underpinnings and practical insights for the research of achievement motivation by confirming that factors such as gender, health, and short-term plans have a significant impact on it. This study departs from the traditional approach of focusing just on the overall level of achievement motivation at the theoretical level. By revealing the internal differentiation characteristics of achievement motivation, it offers a new perspective on this matter and suggests that future theories should place greater emphasis on group heterogeneity. This study offers specific recommendations for higher education practitioners. In order to more effectively boost students’ achievement motivation and consequently improve both academic accomplishment and career development, institutions may create personalized instructional strategies based on specific student characteristics, such as gender, health condition, and short-term plans. Future studies may also use longitudinal tracking designs to investigate the developmental trajectories of achievement motivation, test the cross-regional applicability of the model through multi-regional and multi-institutional comparisons, and use more advanced modeling to investigate possible mediating mechanisms of achievement motivation. This would make it possible to create a developmental model of achievement motivation that is more actionable and explanatory, giving higher education practice a more thorough and scientific theoretical basis.

## Data Availability

The raw data supporting the conclusions of this article will be made available by the authors, without undue reservation.
