# Supplementary material for: Analysis of the current situation of college students’ achievement motivation and influencing factors—an empirical analysis based on a college in Shandong Province
Source: Front Psychol. 2025 Dec 19;16:1636209. doi: 10.3389/fpsyg.2025.1636209 (PMC12757273; doi:10.3389/fpsyg.2025.1636209)
Supplement: Supplementary file 1 [file Supplementary_file_1.docx]

**Appendix S1. Items assignment**

| Items | Assignment |
| --- | --- |
| *Gender* | Male=1；Female=2 |
| Age | 18 years and under=1；over 18 years old=2 |
| Family structure | only child=1；multi-child=0 |
| Living expenses | equal or less than 1000 yuan=1；1001 ~ 1500 yuan=2；  1501 ~ 2000 yuan=3；more than 2000 yuan=4 |
| Family annual income | equal or less than 10,000 yuan =1；30001 ~ 50000 Yuan =2；50001 ~ 80000 Yuan =3；80001 ~ 100000 Yuan =4；100001 ~ 150000 Yuan =5；more than 150,00 yuan =6 |
| Father's literacy level | primary school and below=1；junior high school=2；high school=3；college=4；undergraduate=5；master's degree and above=6 |
| Mother's literacy level | primary school and below=1；junior high school=2；high school=3；college=4；undergraduate=5；master's degree and above=6 |
| Personal health condition | sick=1；not sick=0 |
| Father's health condition | sick=1；not sick=0 |
| Mother's health condition | sick=1；not sick=0 |
| Short-term Plan | no goal=1；graduate without failing a class=2  run for the class committee or student organization=3  work hard to finish school and get a scholarship=4  participate in clubs or other non-major programs=5  participate in competitions in your major=6  Others=7 |
| Future Goals | No direction=1；employment=2；civil servants=3；postgraduate=4；other=5 |
