# Supplementary material for: Analysis of the current situation of college students’ achievement motivation and influencing factors—an empirical analysis based on a college in Shandong Province
Source: Front Psychol. 2025 Dec 19;16:1636209. doi: 10.3389/fpsyg.2025.1636209 (PMC12757273; doi:10.3389/fpsyg.2025.1636209)
Supplement: Supplementary file 2 [file Supplementary_file_2.docx]

**Appendix S2. Chinese version Achievement Motivation Scale**

| 题目 | 完全不符合（1分） | 有些不符合（2分） | 基本符合（3分） | 非常符合（4分） |
| --- | --- | --- | --- | --- |
| 1. 我喜欢新奇的、有困难的任务，甚至不惜冒风险 | 1 | 2 | 3 | 4 |
| 2. 我在完成有困难的任务时，感到快乐 | 1 | 2 | 3 | 4 |
| 3. 我会被那些能了解自己有多大才智的工作所吸引 | 1 | 2 | 3 | 4 |
| 4. 我喜欢尽了最大努力能完成的工作 | 1 | 2 | 3 | 4 |
| 5. 我喜欢对我没有把握解决的问题坚持不懈地努力 | 1 | 2 | 3 | 4 |
| 6. 对于困难的任务，即使没有什么意义，我也很容易 卷进去 | 1 | 2 | 3 | 4 |
| 7. 面对能测量我能力的机会，我感到是一种鞭策和挑 战 | 1 | 2 | 3 | 4 |
| 8. 我会被有困难的任务所吸引 | 1 | 2 | 3 | 4 |
| 9. 对于那些我不能确定是否能成功的工作，最能吸引 我 | 1 | 2 | 3 | 4 |
| 10. 给我的任务即使有充裕的时间，我也喜欢立即开始 做 | 1 | 2 | 3 | 4 |
| 11.能够测量我能力的机会，对我是有吸引力的 | 1 | 2 | 3 | 4 |
| 12. 面临我没有把握克服的难题时，我会非常兴奋，快 乐 | 1 | 2 | 3 | 4 |
| 13. 如果有事不能立刻理解，我会很快对它产生兴趣 | 1 | 2 | 3 | 4 |
| 14. 对我来说，重要的是做有困难的事，即使无人知道 也无关重要 | 1 | 2 | 3 | 4 |
| 15. 我希望把有困难的工作分配给我 | 1 | 2 | 3 | 4 |
| 16. 我讨厌在完全不能确定会不会失败的情境中工作 | 1 | 2 | 3 | 4 |
| 17. 在结果不明的情况下，我担心失败 | 1 | 2 | 3 | 4 |
| 18. 在完成我认为是有困难的任务时，我担心失败 | 1 | 2 | 3 | 4 |
| 19. 一想到要去做那些新奇的、有困难的工作，我就感到 不安 | 1 | 2 | 3 | 4 |
| 20. 我不喜欢那些测量我能力的场面 | 1 | 2 | 3 | 4 |
| 21. 我对那些没有把握能胜任的工作感到忧虑 | 1 | 2 | 3 | 4 |
| 22. 我不喜欢做我不知道能否完成的事，即使别人不知 道也一样 | 1 | 2 | 3 | 4 |
| 23. 在那些测量能力的情境中，我感到不安 | 1 | 2 | 3 | 4 |
| 24. 对需要有特定机会才能解决的事，我会害怕失败 | 1 | 2 | 3 | 4 |
| 25. 那些看起来相当困难的事，我做时很担心 | 1 | 2 | 3 | 4 |
| 26. 我不喜欢在不熟悉的环境下工作，即使无人知道也 一样 | 1 | 2 | 3 | 4 |
| 27. 如果有困难的工作要做，我希望不要分配给我 | 1 | 2 | 3 | 4 |
| 28.我不希望做那些要发挥我能力的工作 | 1 | 2 | 3 | 4 |
| 29. 我不喜欢做那些我不知道我能否胜任的事 | 1 | 2 | 3 | 4 |
| 30. 当我遇到我不能立即弄懂的问题，我会焦虑不安 | 1 | 2 | 3 | 4 |
